# The Association between LIPC rs493258 Polymorphism and the Susceptibility to Age-Related Macular Degeneration

**DOI:** 10.3390/ijerph13101022

**Published:** 2016-10-18

**Authors:** Yafeng Wang, Mingxu Wang, Xiaoqing Zhang, Jing Nie, Ming Zhang, Xiaohong Liu, Le Ma

**Affiliations:** 1 The First Affiliated Hospital of Xi’an Jiaotong University, College of Medicine, Xi’an Jiaotong University, Xi’an 710061, China; wyf.90.25.wyf@stu.xjtu.edu.cn; 2 Key Laboratory of Shaanxi Province for Craniofacial Precision Medicine Research, College of Stomatology, Xi’an Jiaotong University, Xi’an 710004, China; 3 School of Public Health, Xi’an Jiaotong University Health Science Center, Xi’an 710061, China; wangmx601@mail.xjtu.edu.cn; 4 Department of Public Health, Xi’an Medical University, Xi’an 710021, China; dyzhou@nwpu.edu.cn; 5 School of Humanities, Xi’an Jiaotong University, Xi’an 710049, China; boraisrighthere@sina.com; 6 Department of Internal Medicine, Xi’an Honghui Hospital, Xi’an 710054, China

**Keywords:** age-related macular degeneration, LIPC, polymorphism, meta-analysis

## Abstract

The purpose of this study was to evaluate the association of the hepatic lipase (LIPC) rs493258 polymorphism and susceptibility to age-related macular degeneration (AMD). A systematic search in PubMed, EMBASE, and ISI web of science databases was performed to identify eligible published studies without language restrictions up to April 2016. Pooled odds ratios (ORs) with 95% confidence intervals (CIs) in different stages of AMD were estimated under different genetic models using meta-analytic methods. Seven studies comprising 20,559 cases and 17,200 controls met the inclusion criteria and were included in the meta-analysis. The LIPC rs493258 polymorphism showed a significant association with a lower risk of AMD under the allelic model (OR = 0.87, 95% CI = 0.84–0.90). Significant relationships between the variant and AMD were also observed in other genetic models (OR ranging from 0.71 to 0.86, all *p* < 0.05). Stratified analysis based on ethnicity found that LIPC rs493258 polymorphism had a significant association with the decreased risk of the disease in the Caucasian population, but not in the Asian population. For late AMD, significant associations of the rs493258 polymorphism with a lower risk of this disease were also observed in the allelic genetic model (OR = 0.87, 95% CI = 0.83–0.90). This meta-analysis demonstrates that the T allele in the LIPC rs493258 polymorphism was significantly associated with the risk of any and late AMD. The associations of the locus with early and late AMD risk in various populations need further exploration.

## 1. Introduction

With a high incidence rate of 8.7% across the globe, age-related macular degeneration (AMD) is identified as the leading cause of irreversible visual loss among elder individuals in developed countries [1]. It is estimated that, as the population grows, people with AMD will increase to about 288 million in 2040, which could cause an enormous burden for all of society [2]. AMD can be classified into two separate stages based on its clinical characteristics. The pathological hallmark of early AMD is characterized by the accumulation of the lipid protein and its peroxidation products within Bruch’s membrane, and the formation of drusen. As it progresses to the late stage, geographic atrophy (GA) or choroidal neovascularization (CNV) will occur, which can induce severe visual impairment [3].

AMD is a progressive disease caused by a multitude of factors, including environmental and genetic factors. Although many epidemiological studies have linked age, sex, cigarette smoking, alcohol consumption, light exposure, and food habits to the risk of AMD, the pathogenesis of AMD still remains vague [4,5,6]. More and more evidence demonstrated that oxidative stress may also play an important role in the pathogenesis AMD. With the effect of oxidative stress, lipids and phospholipids can be oxidized into peroxidation products, and the accumulation of these metabolites within Bruch’s membrane causes the formation of drusen, which ultimately leads to AMD [7]. The hepatic lipase (LIPC) can encode hepatic triglyceride lipase, an important enzyme in lipid metabolism, which is expressed in the liver, and can catalyze the hydrolysis of oxidized lipids and retard the accumulation of peroxidation lipids in Bruch’s membrane and the progression of AMD. The rs10468017 variant and the rs493258 variant on 15q22, similar to the major SNPs in the promoter region of the LIPC gene, were identified as critically important in the development of AMD. The rs10468017 variant was demonstrated to have a significant association with decreased AMD risk in a recent meta-analysis. Meanwhile, several studies have shown that the LIPC rs493258 variant was related to AMD risk; however, the conclusions varied significantly [8,9]. In addition, the genetic characteristics of AMD varied at different stages and in different races. Whether such discrepancies effect the association between LIPC rs493258 polymorphism and the risk of AMD in those studies remains unclear.

Therefore, we conducted a meta-analysis to evaluate the association between LIPC rs493258 polymorphism and AMD risk. Moreover, stratified analyses were also performed to explore the association at different AMD stages and in different races.

## 2. Materials and Methods

### 2.1. Search Strategy

A comprehensive literature search of PubMed, EMBASE, and the ISI Web of Science databases was conducted to identify the relevant studies. The search strategy was based on a combination of the terms (“hepatic lipase”, “LIPC” or “rs493258”) and (“AMD”, “macular degeneration”, “retinal degeneration”, “retinal neovascularization”, “choroidal neovascularization”, “retinal drusen” or “geographic atrophy”). The searches were limited to human studies with no language restrictions. In addition, we also checked all of the references of relevant reviews and retrieved eligible articles. When data were inadequate for necessary analysis, corresponding authors were contacted, in most cases successfully, for the retrieval of missing data.

### 2.2. Inclusion Criteria and Data Extraction

The included studies fulfilled the following inclusion criteria: (1) the original report evaluated the association of the LIPC rs493258 polymorphism with the risk of early or late AMD; (2) the study design was limited to cohort, case-control, or cross-sectional design; and (3) the study presented available data which provided an odds ratio (OR) with a 95% confidence interval (CI) under different genetic models or sufficient data to estimate them.

The following information was independently extracted from each study included in the present meta-analysis by two investigators (Yafeng Wang and Mingxu Wang): the first author’s last name, year of publication, study design, sample size, the ethnicity of the study population, the mean age, proportion of male, definition of cases and controls, classification criteria, genotype distributions in cases and controls, genotyping methods, and minor allele frequency and Hardy-Weinberg equilibrium (HWE) among controls. The results were compared, and any inconsistencies were resolved through discussion.

### 2.3. Quality Assessment

The quality of the included studies was independently appraised with the nine-item Newcastle Ottawa Quality Scale (NOS), a widely used tool for the quality assessment of observational studies [10]. Three broad perspectives of the included studies—selection, comparability, and exposure—were assessed, and each satisfactory answer received one star. The NOS ranges from zero (worst) to nine stars (best). Studies with a score of five stars or greater were considered to be of high quality, whereas scores less than four were considered to be of low quality [11]. Two investigators (Yafeng Wang and Mingxu Wang) assessed the quality of the included studies independently, and any disagreements were resolved via discussion with a third investigator (Le Ma).

### 2.4. Statistical Analysis

HWE was tested by the chi-square test. The strength of associations between the LIPC rs493258 polymorphism and the risk of AMD was assessed by odds ratios (ORs) with 95% confidence intervals (CIs). We explored the association of the LIPC rs493258 polymorphism with AMD risk under the allelic (T vs. C), dominant ((TT + TC) vs. CC), heterozygous (TC vs. CC), and homozygous (TT vs. CC) genetic models, respectively (T represented minor allele). The Cochran’s Q statistic and *I*^2^ index were employed to assess the between-study heterogeneity. A *p* value < 0.10 for the Q test showed an existence of heterogeneity, and an *I*^2^ value >50% indicated a statistical significance. When heterogeneity was negligible, the fixed-effects model was chosen to evaluate the pooled estimate of risk; otherwise, the random-effects model was performed. Subgroup analysis and meta-regression were performed to explore the potential sources of heterogeneity between studies. In addition, sensitivity analysis by sequentially removing one study at a time was performed to confirm the stability of the results. Publication bias was assessed by the Begg test and the Egger test [12,13]. All statistical analyses were performed using STATA version 11 (StataCorp, College Station, TX, USA). Except where otherwise specified, a *p* value < 0.05 was considered significant.

## 3. Results

### 3.1. Characteristics of the Studies

**Figure 1:**
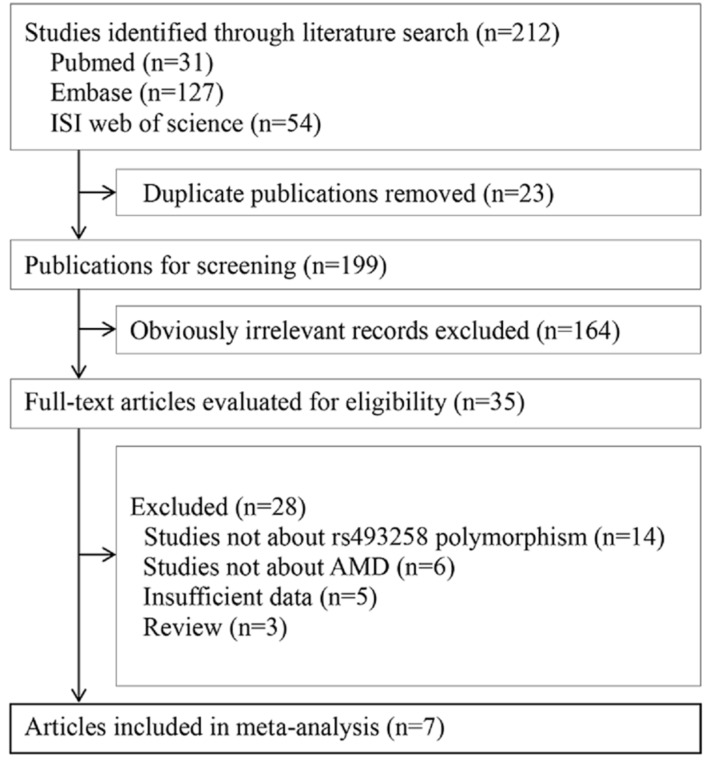
The flowchart of study inclusion and exclusion.

A total of 212 articles were identified by the primary literature search (Figure 1). After the removal of duplicates, 164 articles were excluded based on review of the titles and abstracts. The full text article of the remaining 35 studies was obtained and evaluated according to the eligibility criteria. Finally, seven articles including 17 studies meeting the predetermined inclusion criteria were used for meta-analysis [8,9,14,15,16,17,18].

Characteristics of the included studies are summarized in Table 1. The number of subjects contained 20,559 cases and 17,200 controls. Among the 17 studies, all studies were case-control design; 14 of them were genome-wide association studies (GWAS’s). Fifteen studies were conducted in Caucasians and three in Asians. The mean age ranged from 65.4 to 80.3 in case groups and 44.5 to 77.0 in control groups. Twelve studies used the Clinical Age-Related Maculopathy Staging (CARMS) criteria to establish AMD, whereas three studies used the International Classification and Grading System (ICGS), one used the Wisconsin Age-Related Maculopathy Grading System (WARMGS) criteria, and one study did not report it. All studies were considered to be of high quality. Except for two studies that did not provide the available data, none of the 15 studies showed evidence of departure from HWE (*p* > 0.05).

### 3.2. The LIPC rs493258 Polymorphism and AMD

**Figure 2:**
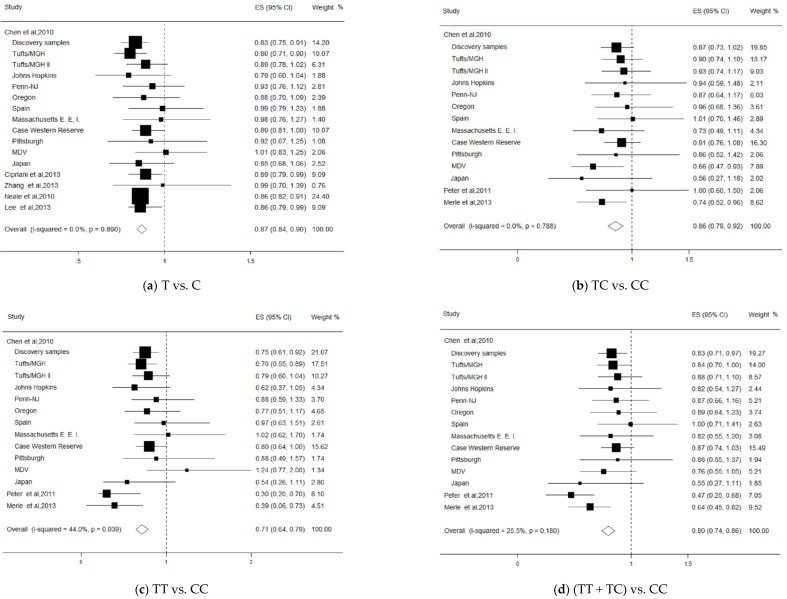
Forest plot on the associations between the LIPC rs493258 polymorphism and age-related macular degeneration (AMD) under the different genetic models. The boxes and lines indicate the odds ratios (ORs) and their 95% confidence intervals (CIs) on a log scale for each study. The pooled odds ratio is represented by a diamond. The size of the black squares indicates the relative weight of each estimate. (**a**) T vs. C; (**b**) TC vs. CC; (**c**) TT vs. CC; (**d**) (TT + TC) vs. CC.

Sixteen studies evaluated the associations between the LIPC rs493258 polymorphism and risk of AMD in the allelic (T vs. C) genetic model, and 14 studies in the other three genetic models. When these studies were pooled in the present meta-analysis, the results showed that the rs493258 variant showed a significant summary OR under an allelic model of T vs. C (OR = 0.87, 95% CI = 0.84–0.90; Figure 2), without significant heterogeneity (*I*^2^ = 0%, *p* = 0.89). The significant relationships between this variant and the AMD risk were also observed in other genetic models (TC vs. CC: OR = 0.86, 95% CI = 0.79–0.92; TT vs. CC: OR = 0.71, 95% CI = 0.64–0.79, (TC + TT) vs. CC: OR = 0.80, 95% CI = 0.74–0.86, Figure 2). The subgroup analysis was performed and the results based on races found that the rs493258 variant had a significant association with the decreased risk of the disease in Caucasians (OR = 0.87, 95% CI = 0.84–0.89), but not in Asians (OR = 0.88, 95% CI = 0.72–1.05, Table 2). The result of the stratified analysis based on study design revealed that the rs493258 variant had a significant association with AMD risk in the GWAS study. The stratified analysis adjusting for CFH showed that the factor did not significantly alter the shape of association. In addition, we performed the sensitivity analysis and found the robustness of the relationship between the rs493258 variant and AMD risk. There was no evidence for the presence of publication bias in the eligible studies as performed by Begg’s funnel plot (*p* = 0.24) and Egger’s regression test (*p* = 0.17).

### 3.3. The LIPC rs493258 Polymorphism and Late AMD

**Figure 3:**
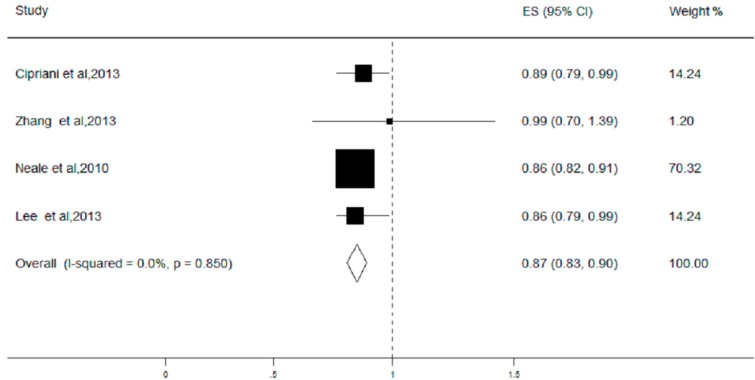
Forest plot on the association between the LIPC rs493258 polymorphism and late age-related macular degeneration (AMD) under the allelic (T vs. C) genetic model. The boxes and lines indicate the odds ratios (ORs) and their 95% confidence intervals (CIs) on a log scale for each study. The pooled odds ratio is represented by a diamond. The size of the black squares indicates the relative weight of each estimate.

We subsequently evaluated the association of the rs493258 variant with late AMD in six studies. Four studies assessed the associations of the rs493258 variant with late AMD in the allelic genetic model, and two studies were in the dominant, heterozygous, and homozygous genetic models. The results found that the T allele in the rs493258 variant significantly decreased the risk of late AMD (OR = 0.87, 95% CI = 0.83–0.90; Figure 3). Compared with the CC genotype, the individuals with the TT and TT + CT genotypes had lower risks of having AMD (TT vs. CC: OR = 0.22, 95% CI = 0.02–0.45; (TT + TC) vs. CC: OR = 0.36, 95% CI = 0.15–0.57). The result of the stratified analysis showed that AMD risk was significantly reduced in Caucasians in the allelic (T vs. C) genetic model (OR = 0.86, 95% CI = 0.83–0.90), but not in the Asian population (OR = 0.99, 95% CI = 0.64–1.34). In addition, the result of the sensitivity analysis, removing each study one at a time, found that the pooled ORs remained stable. Neither Begg’s test nor Egger’s test showed publication bias (*p* > 0.10).

## 4. Discussion

Our meta-analysis found that the LIPC rs493258 polymorphism was significantly associated with a decreased risk of AMD. The stratified analysis based on different races showed that LIPC gene was only significantly protective in Caucasians. The pooled ORs decreased from 0.86 to 0.71 in the heterozygous and homozygous models, suggesting that increasing copy numbers of the T allele was associated with decreased AMD risk.

In 2010, the associations of AMD with several genes implicated in HDL metabolism were reported. Similar to the main HDL-related gene, the LIPC gene has been reported to be significantly associated with AMD in two GWAS’s [9,17]. In the present meta-analysis, our finding also demonstrated that the LIPC rs493258 polymorphism might be a genetic protective factor for AMD.

The growing body of evidence has identified that the accumulation of cellular debris, lipids, and their peroxidation products within Bruch’s membrane results in the formation of drusen [19,20]. These changes in Bruch’s membrane induce the thickening of Bruch’s membrane and the fracture of its elastic fiber layer [21]. Subsequently, it decreases the capacity of the retinal blood circulation, induces tissue hypoxia and the production of angiogenic signaling molecules, and leads to capillary vessel hyperplasia and the formation of choroidal neovascularization, which predominantly results in the progression of AMD [22,23]. In addition, oxidative stress can cause the oxidation of phospholipids. These pro-inflammatory oxidation products can lead to the apoptosis of RPE, the production of VEGF, and endogenous angiogenesis, which ultimately induce the accumulation of lipoprotein within Bruch’s membrane and the formation of drusen [24]. Although the previous study reported that rs493258 might not be the functional variant, the rs493258 variant was found to be in high linkage disequilibrium with the functional variant (rs10468017), which might enhance the LIPC abundance and increase the expression of hepatic triglyceride lipase in the liver. Similar to an important enzyme in the HDL cholesterol pathway of lipid metabolism, hepatic triglyceride lipase can catalyze the hydrolysis of phospholipids, triglycerides, and acyl-CoA thioesters, which can decrease the levels of lipids and affect blood lipid homeostasis [8,9,14,17]. Subsequently, the lipids and peroxidation products, which are locally produced in the RPE instead of being directly deposited from the circulation, would decrease under the effect of the blood-retinal barrier; thus, the accumulation of damaging biomolecules in Bruch’s membrane would be retarded [8,25,26,27]. It would slow down the formation of drusen and the progression to choroidal neovascularization, indicating the association between the LIPC rs493258 polymorphism and a decreased risk of AMD.

The results of the present meta-analysis found that the effect of the LIPC rs493258 polymorphism on AMD might differ in various races. In Caucasians, we observed a strong association between the rs1883025 polymorphism and AMD risk, but we did not find such an association in Asians. Previous studies have shown that the frequency of the T allele varied in two races; and the possible higher T-allele frequency observed in Caucasians could explain the significant association in such races [28]. In addition, from the heterozygous model to the homozygous model, an additional copy of the minor allele could decrease the AMD risk by approximately 15%, which would also support the latter hypothesis. Moreover, the limited number of included studies could cause the large scope of standard deviation, which might account for the insignificant association of this polymorphism with AMD in Asians.

For a proper interpretation of the results, some limitations of our study should be mentioned. First, the pooled results were merely addressed in late stage and overall AMD; the association of early AMD with the LIPC rs493258 polymorphism was not evaluated in the meta-analysis. In addition, the previous studies were based mainly on Caucasians; therefore, the relationship in early AMD and in other ethnic groups, especially in Asians, need to be investigated further. Second, the present analysis was based primarily on unadjusted estimates that did not control confounding factors, including environmental factors (e.g., smoking, alcohol consumption, and food habits) and genetic factors. Thus, further research studies should be conducted if individual data are available, which could adjust other covariates including age, sex, environmental factors, and genetic factors. Third, only published studies were included in our meta-analysis; thus, the potential publication bias was also a concern. Although the apparent publication bias by statistical tests was not observed, we could not completely rule out this problem because a much greater number of studies is necessary in order to detect it adequately.

## 5. Conclusions

The meta-analysis demonstrated the association of LIPC rs493258 polymorphism with AMD. Carriage of the LIPC rs493258 T allele can decrease the risk of developing AMD. Moreover, this polymorphism can decrease genetic susceptibility for AMD in Caucasians. However, only one study has assessed the association of LIPC rs493258 polymorphism with early AMD, and the number of included studies based on Asians was relatively limited; therefore, further well-designed large-perspective cohort studies with interethnic populations are required to evaluate these associations at different stages of AMD.

## Figures and Tables

**Table 1:** Characteristics of studies included in this meta-analysis of the LIPC rs493258 polymorphism and age-related macular degeneration.

Source	Country	Study Design	Sample Size (Case/Control)	Mean Age, y (Case/Control)	Classification Criteria	Type of Case	Genotyping Method	Adjustment *	HWE	Study Quality **
Neale et al., 2010 [14]	USA	GWAS	6768/5943	79.5/74.2	CARMS	Late AMD	iPLEX, AFFY	Yes	Yes	High
Chen et al., 2010 [9]										
Discovery	USA	GWAS	2157/1150	78.6/74.1	CARMS	Any AMD	ILMN	Yes	Yes	High
Tufts/MGH II	USA	GWAS	798/1632	79.7/73.0	CARMS	Any AMD	ILMN	Yes	Yes	High
Tufts/MGH	USA	GWAS	821/1709	80.3/74.1	CARMS	Any AMD	ILMN	Yes	Yes	High
Johns Hopkins	USA	GWAS	641/122	75.5/74.7	CARMS	Any AMD	ILMN	Yes	Yes	High
Penn-NJ	USA	GWAS	556/347	79.8/75.6	CARMS	Any AMD	ILMN	Yes	Yes	High
Oregon	USA	GWAS	509/253	79.8/74.0	CARMS	Any AMD	ILMN	Yes	Yes	High
Spain	Spain	GWAS	348/276	76.7/75.1	CARMS	Any AMD	ILMN	Yes	Yes	High
ME	USA	GWAS	386/190	76.0/75.4	CARMS	Any AMD	ILMN	Yes	Yes	High
CWR	USA	GWAS	1178/1430	78.5/72.5	CARMS	Any AMD	ILMN	Yes	Yes	High
Pittsburgh	USA	GWAS	170/143	69.9/76.7	CARMS	Any AMD	ILMN	Yes	Yes	High
MDV	USA	GWAS	690/245	75.7/68.4	CARMS	Any AMD	ILMN	Yes	Yes	High
Japan	Japan	GWAS	654/333	74.8/74.2	CARMS	Any AMD	ILMN	Yes	Yes	High
Peter et al., 2011 [15]	European	Case-control	146/1269	74.5/73.6	WARMGS	Any AMD and late AMD	TM, AB	No	Yes	High
Cipriani et al., 2012 [16]	UK	GWAS	893/2199	78.6/44.5	ICGS	Late AMD	ILMN	No	NP	High
Merle et al., 2013 [8]	European	Case-control	347/1031	78.3/76.5	ICGS	Any AMD and late AMD	TM, AB	Yes	Yes	High
Zhang et al., 2013 [17]	China	Case-control	157/204	65.4/69.0	NR	Late AMD	TM, AB	Yes	NP	High
Lee et al., 2013 [18]	USA	GWAS	1626/859	79.3/72.6	ICGS	Late AMD	AB	No	Yes	High

AB: Applied Biosystems; AFFY: Affymetrix SNP GeneChip; AMD: age-related macular degeneration; CARMS: Clinical Age-Related Maculopathy Staging System; CIRCL: the Cologne Image Reading Center and Laboratory; GWAS: genome-wide association study; HWE: Hardy-Weinberg equilibrium in controls; ICGS: International Classification and Grading System; ILMN: Illumina Human CNV370v1 Bead Array; MAF: minor allele frequency in control; MALDI-TOF MS: matrix-assisted laser desorption ionization-time-of-flight mass spectrometry; NR: not reported; TM: TaqMan; WARMGS: Wisconsin Age-related Maculopathy Grading System. * Adjust for CFH gene. ** Study quality was judged based on Newcastle-Ottawa Scale.

**Table 2:** Stratified analysis of the association between the LIPC rs493258 polymorphism and age-related macular degeneration in the allelic (T vs. C) genetic model.

Subgroup	N	Cases/Controls	Pooled OR (CI)	*p*	
				Heterogeneity	Meta-Regression
Any AMD	16	20,559/17,200	0.87 (0.84, 0.90)	0.89	
Late AMD	4	11,650/10,307	0.87 (0.83, 0.90)	0.85	
Ethnicity					
Caucasians	14	19,712/16,714	0.87 (0.84, 0.89)	0.83	0.43
Asians	2	847/486	0.88 (0.72, 1.05)	0.49	
Age of case					
≥75	13	18,022/16,469	0.86 (0.83, 0.89)	0.90	0.22
<75	3	1537/731	0.93 (0.80, 1.06)	0.51	
Study design					
GWAS	15	20,355/17,043	0.87 (0.84, 0.89)	0.88	0.76
Case-control	1	204/157	0.99 (0.65, 1.34)		
Adjusting for CFH gene					
Yes	14	15,881/12,993	0.87 (0.83, 0.90)	0.81	0.25
No	2	4678/4207	0.88 (0.80, 0.95)	0.68	
